# Investigating the Transient Regenerative Potential of Cardiac Muscle Using a Neonatal Pig Partial Apical Resection Model

**DOI:** 10.3390/bioengineering9080401

**Published:** 2022-08-18

**Authors:** Katherine M. Copeland, Bryn L. Brazile, J. Ryan Butler, Jim Cooley, Erin Brinkman-Ferguson, Andrew Claude, Sallie Lin, Sammira Rais-Rohani, Bradley Welch, Sara R. McMahan, Kytai T. Nguyen, Yi Hong, Sharan Ramaswamy, Zhi-Ping Liu, Pietro Bajona, Matthias Peltz, Jun Liao

**Affiliations:** 1 Department of Bioengineering, University of Texas at Arlington, Arlington, TX 76010, USA; 2 Department of Biological Engineering, College of Veterinary Medicine, Mississippi State University, Starkville, MS 39762, USA; 3 Department of Biomedical Engineering, Florida International University, Miami, FL 33174, USA; 4 Department of Cardiovascular and Thoracic Surgery, Department of Internal Medicine, University of Texas Southwestern Medical Center, Dallas, TX 75390, USA; 5 Allegheny Health Network-Drexel University College of Medicine, Pittsburgh, PA 15212, USA

**Keywords:** neonatal pig heart, partial apex resection, transient cardiac regeneration, proliferative mesenchymal cells, cardiomyocytes, cardiac fibrosis

## Abstract

Researchers have shown that adult zebrafish have the potential to regenerate 20% of the ventricular muscle within two months of apex resection, and neonatal mice have the capacity to regenerate their heart after apex resection up until day 7 after birth. The goal of this study was to determine if large mammals (porcine heart model) have the capability to fully regenerate a resected portion of the left ventricular apex during the neonatal stage, and if so, how long the regenerative potential persists. A total of 36 piglets were divided into the following groups: 0-day control and surgical groups and seven-day control and surgical groups. For the apex removal groups, each piglet was subjected to a partial wall thickness resection (~30% of the ventricular wall thickness). Heart muscle function was assessed via transthoracic echocardiograms; the seven-day surgery group experienced a decrease in ejection fraction and fractional shortening. Upon gross necropsy, for piglets euthanized four weeks post-surgery, all 0-day-old hearts showed no signs of scarring or any indication of the induced injury. Histological analysis confirmed that piglets in the 0-day surgery group exhibited various degrees of regeneration, with half of the piglets showing full regeneration and the other half showing partial regeneration. However, each piglet in the seven-day surgery group demonstrated epicardial fibrosis along with moderate to severe dissecting interstitial fibrosis, which was accompanied by an abundant collagenous extracellular matrix as the result of a scar formation in the resection site. Histology of one 0-day apex resection piglet (briefly lain on and accidentally killed by the mother sow three days post-surgery) revealed dense, proliferative mesenchymal cells bordering the fibrin and hemorrhage zone and differentiating toward immature cardiomyocytes. We further examined the heart explants at 5-days post-surgery (5D PO) and 1-week post-surgery (1W PO) to assess the repair progression. For the 0-day surgery piglets euthanized at 5D PO and 1W PO, half had abundant proliferating mesenchymal cells, suggesting active regeneration, while the other half showed increased extracellular collagen. The seven-day surgery piglets euthanized at 5D PO, and 1W PO showed evidence of greatly increased extracellular collagen, while some piglets had proliferating mesenchymal cells, suggesting a regenerative effort is ongoing while scar formation seems to predominate. In short, our qualitative findings suggest that the piglets lose the full myocardial regenerative potential by 7 days after birth, but greatly preserve the regenerative potential within 1 day post-partum.

## 1. Introduction

As a leading cause of morbidity and mortality, myocardial infarction (MI) affects more than eight million Americans, with ~620,000 new MI cases each year [1]. MI causes massive heart cell death, scar tissue formation, and a decrease in heart function, leading to poor quality of life and heart failure. Conventional therapeutic strategies for MI include pharmacological treatments, thrombolytic therapies, and reperfusion strategies and are able to improve short-term MI survival [2,3,4,5,6,7,8,9]. However, these strategies do not restore normal cardiac function due to the permanent loss of cardiomyocytes and the poor regenerative potential of the heart muscle. As a promising strategy to revitalize the MI scar tissues, stem cell therapy has gained the attention of many researchers, with the hope to re-build functional cardiomyocytes capable of integrating into the host heart and hence restoring cardiac function [3,4,5,6,7,8,9,10,11]. Unfortunately, the success rate for stem cells to differentiate into cardiomyocytes and the retention of those grafted cells are very low, limiting the potential of cell therapy for cardiac repair and regeneration [3,4,5,6,7,8,9,10,11,12].

The underlying mechanisms causing the low efficiency of cardiac differentiation and poor cell retention remain unclear. Some researchers have shown that chemical and biological factors, such as poor oxygen supply, deprived nutrient condition, reactive oxygen species, and inflammatory cytokines, were potential culprits for the low efficiency of cardiac differentiation [3,4,5,6,7,8,9,10,11,12]. Moreover, the biophysical and mechanical microenvironment where the cells situate, such as extracellular matrix (ECM) composition [13,14,15,16,17,18], ultrastructure [13,19,20], and stiffness [21,22,23,24,25,26,27,28,29], could affect stem cell differentiation via cell-ECM interaction pathways. How to create a favorable condition/microenvironment that overcomes the above-mentioned obstacles and, at the same time, provides the needed biochemical, cellular, and biophysical cues for cardiac regeneration is thus an important question to answer.

When facing formidable challenges in medicine and bioengineering, people often seek help from nature. Observing successful examples of tissue regeneration, specifically in hearts, and uncovering the underlying mechanisms hence offer hope for identifying optimal cardiac reparative and regenerative strategies by replicating those enabling conditions/mechanisms. Researchers have reported that less complex organisms, such as fish and amphibians, retain the ability to regenerate through cell proliferation throughout their lifetime. More complex organisms, such as humans, lose this ability almost immediately after birth [30,31,32]. Researchers hence have investigated the regenerative potential of the heart and the underlying mechanisms in in vivo settings using zebrafish and mice as animal models [33,34,35,36,37].

Poss et al. discovered that adult zebrafish have the capacity to regenerate up to 20% of the ventricular muscle within two months of apex resection (20% of the ventricle at the apex was removed by scissors, and a Kimwipe was used to blot the incision, as the wounds bled profusely for 15–45 s before beginning to clot) [33]. Zebrafish can create a new ventricular wall via cardiomyocyte proliferation [33]. Recently, Porrello et al. found that there was a regenerative response in neonatal mice before day 7 after birth that enables cardiac tissue repair similar to that of the zebrafish [35]. Porrello found that neonatal mice can regenerate their hearts after full apex resection (scissor resecting until the left ventricular chamber was exposed [35]) until the seventh day after birth via cardiomyocyte proliferation. The majority of the new cardiomyocytes originated from preexisting cardiomyocytes, as indicated by genetic fate mapping [35]. These findings were challenged by Anderson et al. in 2014, who attempted to replicate the study. Anderson found that the neonatal mouse hearts (left ventricle chamber exposure was used as a landmark for resection, and immediate blood clotting sealed the heart and prevented the use of suturing [36]) healed by scar formation and exhibited reduced cardiomyocyte proliferation after full apical resection.

The great importance of cardiac muscle regeneration and the ongoing investigations of this phenomenon in the zebrafish and mouse models warrant more exploration. It is well known that human cardiomyocytes have a limited regenerative capacity, with less than 50% of cardiomyocytes replaced during a normal human lifespan [38,39]. Similarly, mammalian cardiac regeneration declines rapidly after birth, continuously receding with aging [40,41]. The adult mammalian heart initiates an acute MI-associated inflammatory response following injury, often leading to chronic, irreversible pathological remodeling of the heart and compromised cardiac function. In other words, even though adult mammals might replace a small percentage of damaged cardiomyocytes, this regenerative potential is not sufficient to revitalize the heart after a major injury.

The full regeneration of resected apex in zebrafish and neonatal mice studies raises questions regarding myocardial regeneration in large mammals [33,34,35,36,37,42,43,44]. We hence chose a neonatal pig apex resection model to assess the transient regenerative potential of cardiac muscle. The pig heart has been widely used for cardiac research due to the great anatomical and physiological similarities with the human heart [45,46,47]. The similar size, anatomy, and right-dominant coronary vasculature of the pig heart provide a more accurate representation of the human heart than other animal models [45,46,47,48]. Our ultimate goal is to determine if large mammalian animal models have the capability to fully regenerate heart muscle following partial apical resection without the formation of a scar and characterize the timeframe in which this phenomenon occurs.

## 2. Materials and Methods

### 2.1. Pig Model for Apex Partial Resection and Regeneration

The animal experiment protocol was approved by the Mississippi State University Institutional Animal Care and Use Committee (IACUC, #15-045) and was performed in accordance with the National Institutes of Health (NIH, Bethesda, MD, USA) guidelines. For this study, we utilized 36 piglets that were delivered by three mother sows (Yorkshire), which were divided into two study groups, i.e., 0-day-old group (<24 h, N = 17) and seven-day-old group (N = 19) (Table 1 and Supplementary Materials). We chose 0-day and 7-day as two major time points in order to compare with the mouse apex resection study [35]. Each age group was further categorized as the apex removal group (N = 28 total, < 24-h N = 13, 7-day N = 15) and the control group (N = 8 total, <24-h N = 4, 7-day N = 4) (Table 1). In the apex removal group, each piglet was subjected to a thoracotomy and pericardiotomy followed by a partial thickness apical resection. In the control group, the piglets only experienced a thoracotomy and a pericardiotomy (sham surgery). The assignment of groups was randomly implemented. The piglets in both 0-day and seven-day groups were euthanized and necropsied at five days, one week, or four weeks post-operatively (Table 1).

### 2.2. Surgical Procedures

Piglets were anaesthetized with a mixture of 95% oxygen and 5.0% isoflurane, then intubated with a size 3 or 3.5 cuffed endotracheal tube. A heated operating pad maintained the piglet’s body temperature during the procedure. Positive-pressure ventilation was established, and anesthesia was maintained with a mix of 98.0% oxygen and 2.0% isoflurane throughout the surgery. A left thoracotomy was performed through the 4th intercostal space, and ribs were retracted, followed by a pericardiotomy. In the apex removal group, each piglet was subjected to a partial wall thickness resection (~30% of the ventricular wall thickness, located at the apex of the heart). Apical resection was more limited compared to previously described murine studies [35,36] due to the risk of exsanguination with ventricular entry in the larger animal. (We experienced two piglet losses during the surgical procedure where the left ventricle was entered during the excision). Following resection, the chest cavity was immediately closed, and the piglets were given subcutaneous buprenorphine injection for post-operative pain management. At the end of the experiments, piglets were euthanized via intra-cardiac injection of saturated potassium chloride solution (KCl), which arrests the heart in diastole.

### 2.3. In Vivo Analysis of Heart Function: Echocardiogram

Heart muscle function was assessed via transthoracic echocardiograms for each group. Two-dimensional short axis views of the left ventricle and M-mode tracings were recorded through anterior and posterior left ventricular walls at the papillary muscle level to measure the end-diastolic dimensions (EDD) and the end-systolic dimensions (ESD). The echocardiograms were performed for each piglet before surgery and these subsequent time points: one day post-surgery, five days post-surgery, one week post-surgery, two weeks post-surgery, three weeks post-surgery, and four weeks post-surgery. Specifically, we assessed the differences in ejection fraction and fractional shortening at the above-mentioned time points, as well as between the sham and the apex removal groups. Ejection fraction is the percentage of blood ejected during systole. It is a volumetric calculation based on linear measurements and an assumed shape of the left ventricle during systole and diastole. Fractional shortening is the change in left ventricular lumen diameter in diastole and systole and is calculated by: *(EDD − ESD)/(EDD)*. Fractional shortening is a linear measurement; therefore, it does not assume volume. Both values are expressed as percentages.

### 2.4. Gross Anatomy and Histology

The gross anatomical assessment was performed upon necropsy of each piglet. We explanted the hearts and dissected them into two halves along the echo plane section of the long axis. One half was submerged in 2% paraformaldehyde fixative for 72 h for histological analysis with Masson’s Trichrome and Hematoxylin and Eosin (H & E) staining to determine the potential degree of heart regeneration and other histologic changes at the resection location.

### 2.5. Statistical Analysis

Data were stored and analyzed using RStudio (R Foundation for Statistical Computing, Vienna, Austria). Mean ± standard deviation (STDEV) was used to present the echocardiogram data, including fractional shortening and ejection fraction. Statistical analyses were performed to determine if the parameters were statistically different using one-way analysis of variance (ANOVA). Additionally, Tukey’s test was utilized for post-hoc pair-wise comparisons between the groups. Data were considered significant when *p* < 0.05.

## 3. Results

### 3.1. Surgical Outcomes

**Figure 1:**
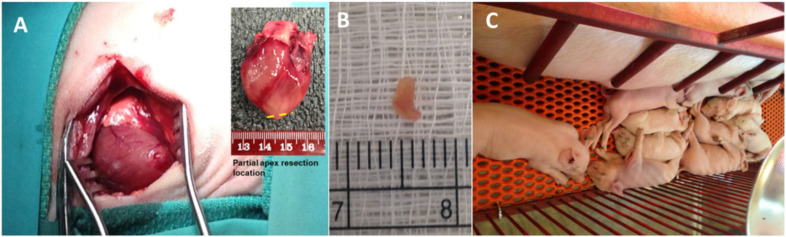
(**A**) Partial apex resection surgery performed on neonatal piglets. Inserted image schematically shows the partial apex resection location. (**B**) Heart muscle tissue resected from the apex of a piglet heart. (**C**) Piglets resting with mother sow after surgery.

The partial apex resection was successfully performed on the 0-day-old and seven-day-old neonatal piglets (Figure 1A,B). For the 0-day group, the average mass of the removed tissue weighed 8.47 ± 11.20 mg, whereas the average mass removed for the seven-day group weighed 5.62 ± 1.75 mg (Table 2). During surgery, it was noted that the 0-day-old hearts were more pliable and could be more easily positioned for the apex resection than the seven-day-old hearts, which caused a higher average resection mass in 0-day surgery group. Moreover, in the seven-day group, we noticed that the apex tip was the thinnest part of the heart due to anatomical development. Therefore, we had to move our resection site slightly anterolateral to the apex on the left ventricular free wall to avoid entry into the ventricular chamber and exsanguination. All piglets handled the surgery well and were able to walk and nurse within minutes of being placed back into the farrowing crate with the mother sow (Figure 1C).

### 3.2. Echocardiogram Results

**Figure 2:**
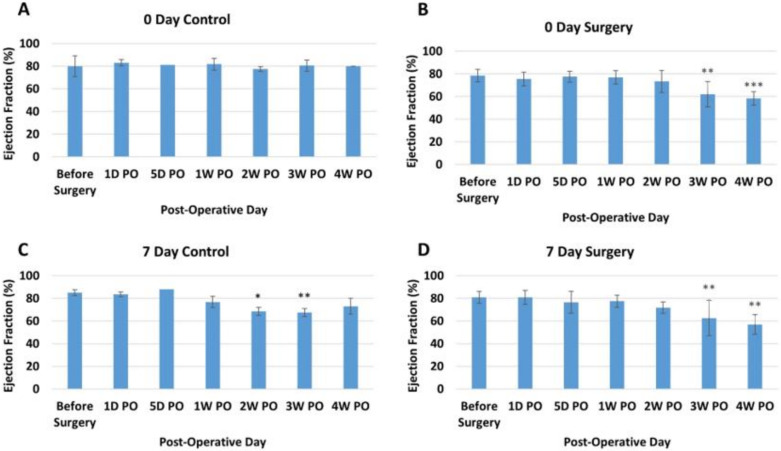
Average ejection fraction (percentage) at different time points for each group. (**A**) Zero-day control group. (**B**) Zero-day surgery group. (**C**) seven-day control group. (**D**) seven-day surgery group. *, **, and *** represent a significant difference (*p* < 0.05) between before surgery and the labeled time point.

***Ejection Fraction***. Ejection fraction (EF) by echocardiography showed no overall trend for the 0-day control group (Figure 2A). The 0-day surgery group, on the other hand, showed a decrease in ejection fraction for the 3rd and 4th weeks post-operation animals (PO) (Figure 2B). The post-hoc comparison results indicated that there was a significant difference between before surgery (78.43 ± 5.6%) and 3W PO (62.0 ± 11.2%) and 4W PO (58.3 ± 6.0%) for the 0-day surgery group (*p* < 0.05). While the EF for the seven-day control group showed no obvious overall trend (Figure 2C), the seven-day surgery group also experienced a significant overall decreasing trend in EF over the course of the study (Figure 2D, *p* < 0.001). The post-hoc comparison results indicated that there was a significant difference (*p* < 0.05) between the before surgery (80 ± 5.21%) and the 3W PO (63 ± 15.50%) and 4W PO (57 ± 8.72%) groups for the seven-day surgery piglets.

**Figure 3:**
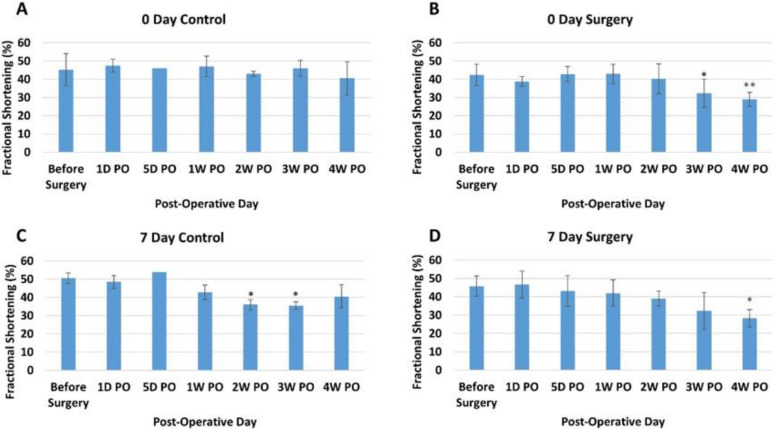
Average fractional shortening (percentage) at different time points for each group. (**A**) Zero-day control group. (**B**) Zero-day surgery group. (**C**) Seven-day control group. (**D**) Seven-day surgery group. * and ** represent a significant difference (*p* < 0.05) between before surgery and the labeled time point.

***Fractional Shortening***. The fractional shortening (FS) assesses the degree of shortening of the left ventricular diameter between end-diastole and end-systole, providing another estimate of systolic function. For our study, the 0-day control piglets showed no overall trend (Figure 3A). Like EF measurements, the 0-day surgery group showed a decreasing trend, which only occurred in the 3rd and 4th weeks PO (Figure 3B). The before surgery and one-day post-operative FS measurements for all the 0-day-old piglets were also within the previously reported range [49]. The FS for the seven-day control group showed no consistent trend, with 2W and 3W decreasing but 4W increasing (Figure 3C). On the other hand, the seven-day surgery group experienced a significant overall decrease in FS over the course of the study (*p* < 0.001) (Figure 3D). The post-hoc comparison results indicated that there was a significant difference between the before surgery time point (45.8 ± 5.48%) and the 4W PO time point (28.3 ± 4.73%) for the seven-day surgery group. This significant drop in FS was larger than the decrease seen in the 0-day surgery piglet (Figure 3B).

### 3.3. Gross Anatomy and Histology Results

**Figure 4:**
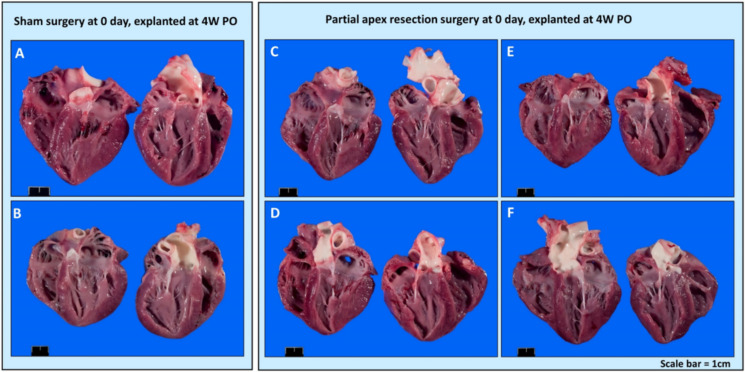
Gross anatomy photos. (**A**) Zero-day control (sham) heart from piglet #2033. (**B**) Zero-day control (sham) heart from piglet #2038. (**C**) Zero-day surgery heart from piglet #2034. (**D**) Zero-day surgery heart from piglet #2035. (**E**) Zero-day surgery heart from piglet #2036. (**F**) Zero-day surgery heart from piglet #2037. Scale bar = 1 cm.

**Figure 5:**
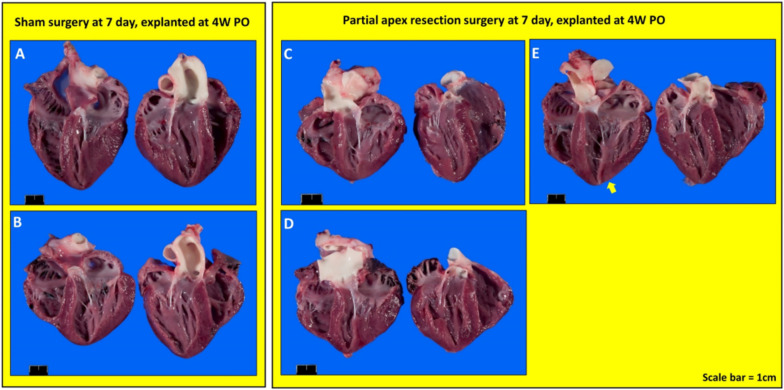
Gross anatomy photos. (**A**) Seven-day control (sham) heart from piglet #2071. (**B**) Seven-day control (sham) heart from piglet #2077. (**C**) Seven-day surgery heart from piglet #2073. (**D**) Seven-day surgery heart from piglet #2075. (**E**) Seven-day surgery heart from piglet #2076. Scale bar = 1 cm.

***Gross Necropsy***. Upon euthanasia, body weight and heart mass were measured for all piglets (Table 3). The piglets in the control and surgery groups were either five days old (0-day surgery group, 5D PO), one week old (0-day surgery group, 1W PO), 12 days old (seven-day surgery, 5D PO), two weeks old (seven-day surgery group, 1W PO), four weeks old (0-day surgery group, 4W PO), or five weeks (5W) (seven-day surgery group, 4W PO) old at the time of euthanasia. Upon gross necropsy, the 0-day surgery hearts (4W PO) showed no signs of scarring or any indication of the induced injury (Figure 4C–F). Of the seven-day surgery hearts (4W PO), one piglet (#2076) in the surgery group had a zone of localized pallor in the myocardium at the site of a pericardial adhesion (possible necrosis, regeneration, or fibrosis) (arrow in Figure 5E). Each of the other hearts in the 7-day surgery groups also had signs of pericardial adhesions.

### 3.4. Histology

#### 3.4.1. Comparing the Outcomes of 0-Day Surgery and Seven-Day Surgery in 4W PO Heart Explants

**Figure 6:**
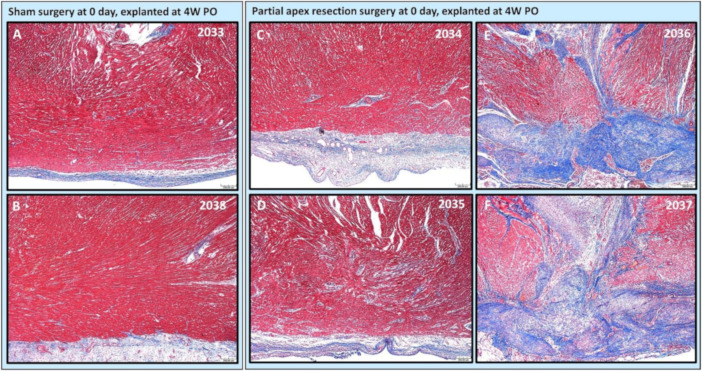
Partial apex resection surgery at 0 days and heart explanted at 4W PO-Masson’s Trichrome histology of 0-day control hearts and 0-day surgery hearts. Heart explants were obtained four weeks post-operation (4W PO). (**A**) Zero-day control (sham) heart from piglet #2033. (**B**) Zero-day control (sham) heart from piglet #2038. (**C**) Zero-day surgery heart from piglet #2034. (**D**) Zero-day surgery heart from piglet #2035. (**E**) Zero-day surgery heart from piglet #2036. (**F**) Zero-day surgery heart from piglet #2037. Histological images were taken at 4× magnification (Scale bar = 200 μm). Masson’s Trichrome: red–muscle; blue–collagen.

**Figure 7:**
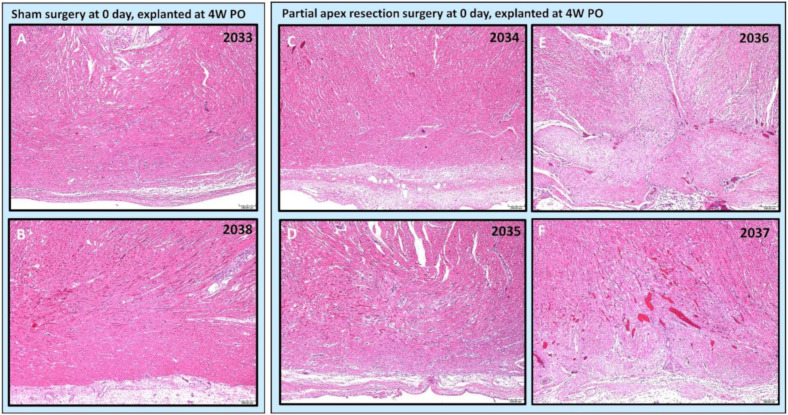
Partial apex resection surgery at 0 days and heart explanted at 4W PO-Hematoxylin and Eosin (H & E) histology of 0-day control hearts and 0-day surgery hearts. Heart explants were obtained four weeks post-operation (4W PO). (**A**) Zero-day control (sham) heart from piglet #2033. (**B**) Zero-day control (sham) heart from piglet #2038. (**C**) Zero-day surgery heart from piglet #2034. (**D**) Zero-day surgery heart from piglet #2035. (**E**) Zero-day surgery heart from piglet #2036. (**F**) Zero-day surgery heart from piglet #2037. Histological images were taken at 4× magnification (Scale bar = 200 μm). H & E: pink–cytoplasm; blue–nuclei; intense red–erythrocytes.

***Partial apex resection surgery at 0 days and heart explanted at 4W PO***. Masson’s Trichrome (Figure 6A,B) and H & E (Figure 7A,B) stained images at 4× magnification were taken from the piglet hearts (#2033 and #2038) in the 0-day control group. In these images, there is a clear epicardial layer surrounding the apex region (Figure 6A,B and Figure 7A,B). The myocardium of these hearts shows the normal histology, with central nuclei and elongated sarcoplasm in linear profile (Figure 6A,B and Figure 7A,B).

The histological study revealed that two piglets (#2034 and #2035; Figure 6C,D and Figure 7C,D) in the 0-day surgery group only had subtle deviations from the surrounding myocardium at the resection site. This includes a slight disorganization, some variation in myofiber width, zones of apparent hypercellularity and mild vascular prominence in one pig (#2035; Figure 6D), in the absence of increased extracellular collagenous matrix as determined by Masson’s Trichrome staining. The other piglet (#2034; Figure 6C) had imperceptible changes with a similar absence of increased extracellular collagen, and only mildly increased epicardial fibrosis. The histology of those two piglets (Figure 6 and Figure 7 #2034 and #2035) suggests full regeneration of the resected apex tissue.

The other two piglets (#2036 and #2037; Figure 6E,F) in the 0-day surgery group had a population of proliferative cells that had variable extension into the myocardium and dissection into adjacent cardiac myofibers at 4× magnification. These cells are reminiscent of cardiomyocytes in H & E-stained images (#2036 and #2037; Figure 7E,F), but are associated with abundant extracellular collagenous matrix based on Masson’s trichrome stained images (Figure 6E,F). These images suggest a partial regeneration of the resected apex tissue (#2036 and #2037; Figure 6E,F and Figure 7E,F).

**Figure 8:**
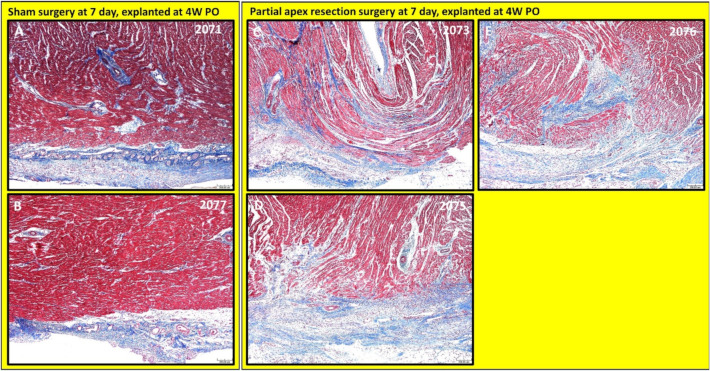
Partial apex resection surgery at seven days and heart explanted at 4W PO-Masson’s Trichrome histology of seven-day control hearts and seven-day surgery hearts. Heart explants were obtained four weeks post-operation (4W PO). (**A**) Seven-day control (sham) heart from piglet #2071. (**B**) Seven-day control (sham) heart from piglet #2077. (**C**) Seven-day surgery heart from piglet #2073. (**D**) Seven-day surgery heart from piglet #2075. (**E**) Seven-day surgery heart from piglet #2076. Histological images were taken at 4× magnification (Scale bar = 200 μm). Masson’s Trichrome: red–muscle; blue–collagen.

**Figure 9:**
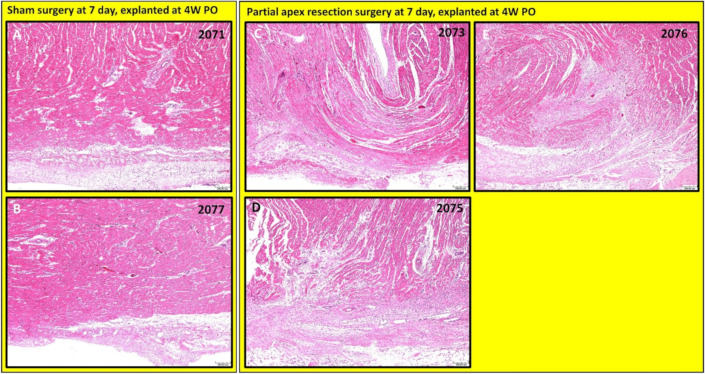
Partial apex resection surgery at seven days and heart explanted at 4W PO-Hematoxylin and Eosin (H & E) histology of seven-day control hearts and seven-day surgery hearts. Heart explants were obtained four weeks post-operation (4W PO). (**A**) Seven-day control (sham) heart from piglet #2071. (**B**) Seven-day control (sham) heart from piglet #2077. (**C**) Seven-day surgery heart from piglet #2073. (**D**) Seven-day surgery heart from piglet #2075. (**E**) Seven-day surgery heart from piglet #2076. Histological images were taken at 4× magnification (Scale bar = 200 μm). H & E: pink–cytoplasm; blue–nuclei; intense red–erythrocytes.

***Partial apex resection surgery at seven days and heart explanted at 4W PO.*** Masson’s Trichrome (Figure 8A,B) and H & E (Figure 9A,B) stained images at 4× magnification were taken from piglet hearts (#2071 and #2077) in the seven-day control group. These control images were almost identical to the control images from the 0-day control group, with a clear epicardial layer overlying the myocardium. The myocardium of these hearts has clear central nuclei and elongated cytoplasm. Slightly more interstitial ECM can be seen in piglet #2071 than in the other control piglet; this is primarily due to the increased vascularization in the image (Figure 8A).

The histological study revealed that each piglet (#2073, #2075, and #2076; Figure 8C–E and Figure 9C–E) in the seven-day surgery group demonstrated apparent epicardial fibrosis along with moderate to severe dissecting interstitial fibrosis, which was accompanied by an abundant collagenous extracellular matrix as seen in the Masson trichrome stained images (Figure 8C–E) and the H & E-stained images (Figure 9C–E) at 4× magnification. This largely increased amount of collagenous ECM is the result of scar formation in the resection site and suggests the piglet hearts lost the full regenerative potential by seven days after birth.

#### 3.4.2. Assessing the Progression of Repair of the Heart Explants at 5D PO and 1W PO

**Figure 10:**
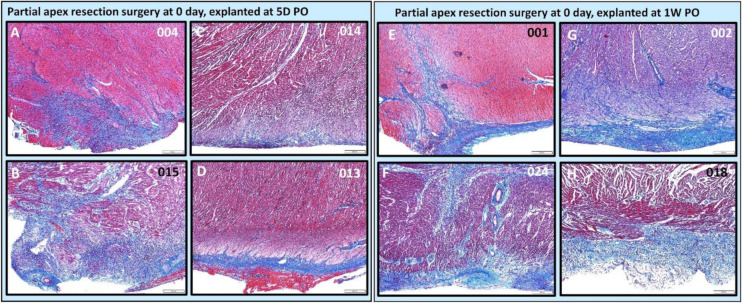
Partial apex resection surgery at 0 day and heart explants at 5D PO and 1W PO-Masson’s Trichrome histology of 0-day surgery heart explants obtained five days post-operation (5D PO): (**A**) Zero-day surgery heart from piglet #004. (**B**) Zero-day surgery heart from piglet #015. (**C**) Zero-day surgery heart from piglet #014. (**D**) Zero-day surgery heart from piglet #013. Masson’s Trichrome histology of 0-day surgery heart explants obtained one-week post-operation (1W PO): (**E**) Zero-day surgery heart from piglet #001. (**F**) Zero-day surgery heart from piglet #024. (**G**) Zero-day surgery heart from piglet #002. (**H**) Zero-day surgery heart from piglet #018. Histological images were taken at 4× magnification (Scale bar = 200 μm). Masson’s Trichrome: red–muscle; blue–collagen.

**Figure 11:**
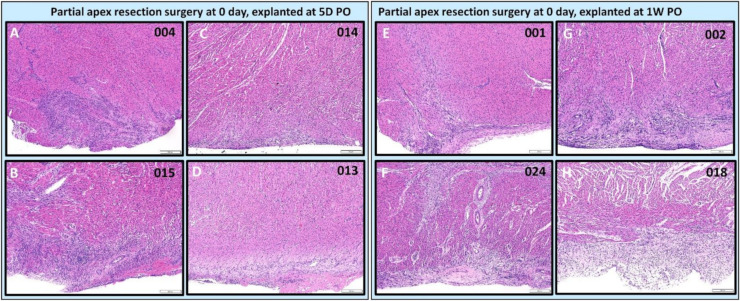
Partial apex resection surgery at 0 day and heart explants at 5D PO and 1W PO-Hematoxylin and Eosin (H & E) histology of 0-day surgery heart explants obtained five days post-operation (5D PO): (**A**) Zero-day surgery heart from piglet #004. (**B**) Zero-day surgery heart from piglet #015. (**C**) Zero-day surgery heart from piglet #014. (**D**) Zero-day surgery heart from piglet #013. H & E histology of 0-day surgery heart explants obtained one-week post-operation (1W PO): (**E**) Zero-day surgery heart from piglet #001. (**F**) Zero-day surgery heart from piglet #024. (**G**) Zero-day surgery heart from piglet #002. (**H**) Zero-day surgery heart from piglet #018. Histological images were taken at 4× magnification (Scale bar = 200 μm). H & E: pink–cytoplasm; blue–nuclei; intense red–erythrocytes.

**Figure 12:**
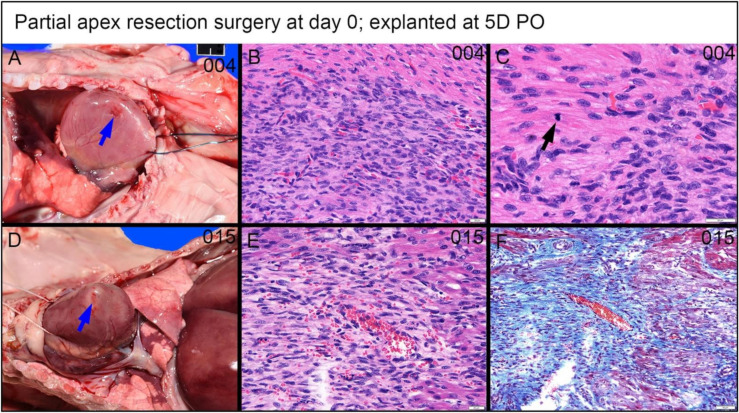
Partial apex resection surgery at 0 days and heart explanted at 5D PO (**A**–**C**)—(**A**) Gross necropsy heart obtained five days post-operation (5D PO) from piglet #004. Note partially filled surgical defect (blue arrow) (Scale bar = 1 cm). (**B**) Hematoxylin and Eosin (H & E) histology (piglet #004) reveals a densely packed population of streaming cells in register with peripheral myocardium and with similar slightly larger elongate often plump nuclei (Scale bar = 20 μm). (**C**) H & E histology (piglet #004) of the marginal zone of proliferative cells has a mitotic figure of a cell identifiable as a cardiomyocyte based on straplike morphology, eosinophilic cytoplasm, and cross striations (black arrows) (Scale bar = 20 μm). Partial apex resection surgery at 0 days and heart explanted at 5D PO (**D**–**F**)—(**D**) Gross necropsy heart obtained five days post-operation (5D PO) from piglet #015. Note partially filled surgical defect (blue arrow) (Scale bar = 1 cm). (**E**) H & E histology (piglet #015) reveals a population of similar streaming elongate cells with elongate nuclei. (**F**) Masson’s trichrome stain (piglet #015) reveals a moderate intervening collagenous matrix.

***Partial apex resection surgery at 0 days and heart explants at 5D PO and 1W PO***. Of the 0-day piglets euthanized 5D PO, Pig 004 and 015 (Figure 10A,B, Figure 11A,B, and Figure 12B,C,E,F) both have a focus of densely proliferative cells extending deeply into the myocardium, consistent with the progression of tissue regeneration or repair. In piglet #004, these cells have plump elongate nuclei and occur in streaming arrays in parallel with adjacent myocardium (Figure 12B). Mitotic figures in cells morphologically identifiable as cardiomyocytes occur in the marginal zone of proliferative cells (Figure 12C, arrow). Coexisting with these mesenchymal cells, the matrix is stained predominately with collagen. Piglet 014 (Figure 10C and Figure 11C) has a shallow focus of mesenchymal cells, including a small amount of ECM. Piglet 013 (Figure 10D and Figure 11D), unlike the other piglets, exhibited signs of epicardial fibrosis that appears as reactive fibrovascular tissue organizing superficial epicardial fibrin.

**Figure 13:**
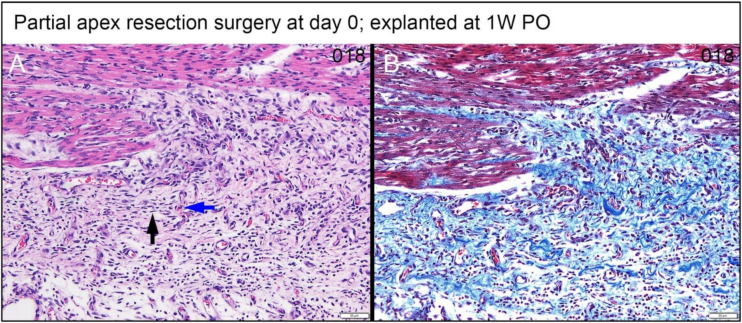
Partial apex resection surgery at 0 days and heart explanted at 1W PO-(**A**) Hematoxylin and Eosin (H & E) histology 0-day surgery heart explant obtained one week post-operation (1W PO) from piglet #018. Higher magnification of the field of Figure 11H reveals proliferative spindle cells with thin nuclei often oriented in stacked arrays (black arrow) that are perpendicular to capillaries (blue arrow) typical of fibrovascular tissue. The relationship to the myocardium is abrupt. (**B**) Masson’s Trichrome of the same field reveals prominent interspersed collagen (Scale bar = 50 μm).

For the 0-day piglets euthanized at 1W PO, two had signs of proliferating mesenchymal cells that suggested active regeneration (#001 & #024; Figure 10E,F and Figure 11E,F), while the other two had an increase in extracellular collagen (#002 & #018; Figure 10G,H and Figure 11G,H). Again, piglet #001 had evidence of regeneration, as well as extracellular matrix (Figure 10E and Figure 11E). This could reflect early signs of cardiac regeneration, i.e., in the lesion region, the cellular mesenchymal and proliferative cells are restoring the heart muscle cells while competing with the trend of extracellular matrix deposition. On the other hand, piglets #002 and #018 (Figure 10G,H and Figure 11G,H) had more fibrovascular tissue extending into the myocardium, suggesting nascent scar formation (Figure 13).

**Figure 14:**
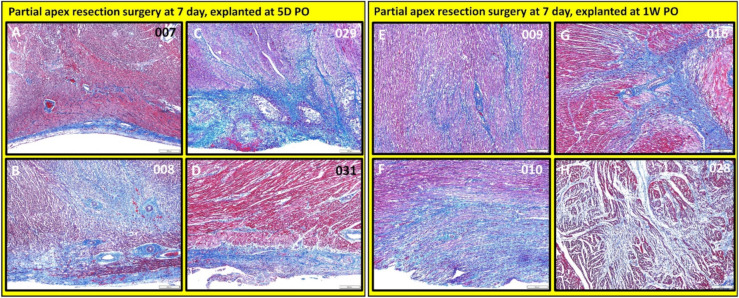
Partial apex resection surgery at seven days and heart explants at 5D PO and 1W PO-Masson’s Trichrome histology of seven-day surgery heart explants obtained five days post-operation (5D PO): (**A**) Seven-day surgery heart from piglet #007. (**B**) Seven-day surgery heart from piglet #008. (**C**) Seven-day surgery heart from piglet #029. (**D**) Seven-day surgery heart from piglet #031. Masson’s Trichrome histology of seven-day surgery heart explants obtained one week post-operation (1W PO): (**E**) Seven-day surgery heart from piglet #009. (**F**) Seven-day surgery heart from piglet #010. (**G**) Seven-day surgery heart from piglet #016. (**H**) Seven-day surgery heart from piglet #028. Histological images were taken at 4× magnification (Scale bar = 200 μm). Masson’s Trichrome: red–muscle; blue–collagen.

**Figure 15:**
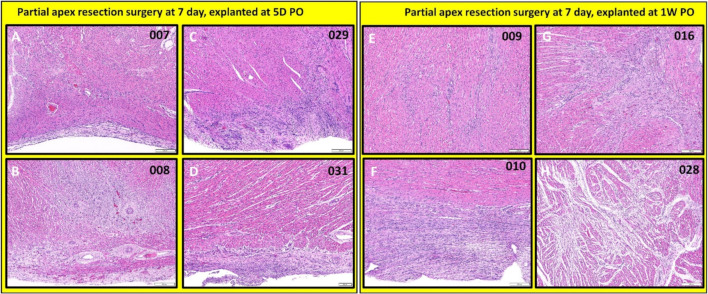
Partial apex resection surgery at seven days and heart explants at 5D PO and 1W PO-Hematoxylin and Eosin (H & E) histology of seven-day surgery heart explants obtained five days post-operation (5D PO): (**A**) Seven-day surgery heart from piglet #007. (**B**) Seven-day surgery heart from piglet #008. (**C**) Seven-day surgery heart from piglet #029. (**D**) Seven-day surgery heart from piglet #031. H & E histology of seven-day surgery heart explants obtained one-week post-operation (1W PO): (**E**) Seven-day surgery heart from piglet #009. (**F**) Seven-day surgery heart from piglet #010. (**G**) Seven-day surgery heart from piglet #016. (**H**) Seven-day surgery heart from piglet #028. Histological images were taken at 4× magnification (Scale bar = 200 μm). H & E: pink–cytoplasm; blue–nuclei; intense red–erythrocytes.

***Partial apex resection surgery at seven days and heart explants at 5D PO and 1W PO.*** The seven-day surgery piglets euthanized at 5D PO (#007, #008, #209, #031; Figure 14A–D and Figure 15A–D) showed mild to severe epicardial fibrosis extending into the myocardium. We did notice that one piglet (#008; Figure 14B and Figure 15B) had a focus of proliferative mesenchymal cells. All the surgical seven-day piglets euthanized at 1W PO (#009, #010, #016, #028; Figure 14E–H and Figure 15E–H) had evidence of greatly increased extracellular collagen. Interestingly, some piglets had evidence of either mesenchymal cells (#010 & #028; Figure 14F,H and Figure 15F,H) or proliferating spindle cells streaming with cardiomyocytes (#016; Figure 14G and Figure 15G), implicating a possible regenerative effort is ongoing while scar formation predominates. It is worthy to note that, although piglets #010, #016, #028 (Figure 14F–H and Figure 15F–H) have proliferative cells, they are loosely arrayed and randomly oriented with scant cytoplasm.

#### 3.4.3. Dense, Proliferative Mesenchymal Cells Bordering the Fibrin and Hemorrhage Zone and Differentiating toward Immature Cardiomyocytes-3D PO Explant after 0-Day Resection Surgery

**Figure 16:**
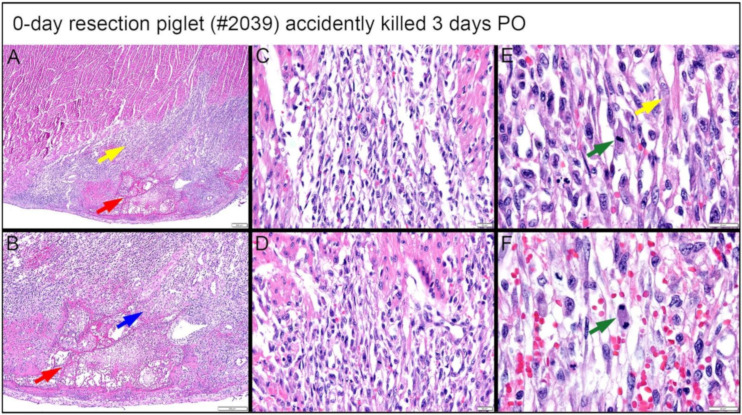
Histological images stained with H & E for the 0-day piglet (#2039) that was accidentally killed by the mother sow three days post apex resection surgery. (**A**) Image of 0-day piglet heart at 2× magnification; (**B**) Image of 0-day piglet heart at 4× magnification; the yellow arrow is the border zone of the dense cellular population; the red arrow is fibrin zone/fibrin; the blue arrow is necrotic cardiomyocytes; scale bar = 200 μm. (**C**) Image of 0-day piglet heart at 20× magnification; (**D**) Image of 0-day piglet heart at 20× magnification; scale bar = 20 μm. (**E**) Image of 0-day piglet heart at 40× magnification; (**F**) Image of 0-day piglet heart at 40× magnification; the green arrow is cells with mitotic events; the yellow arrow indicates the cells resembling immature cardiomyocytes; scale bar = 20 μm. H & E: pink–cytoplasm; blue–nuclei; intense red–erythrocytes.

A piglet (#2039; Figure 16) from the 0-day surgery group was briefly lain on and accidentally killed by the mother sow three days PO. Subsequent to rapid discovery, the piglet was necropsied in fresh condition. Note that the following observed proliferate cells and repairing events at the lesion site are not immediate changes that are caused by suffocation. Upon histological evaluation, the piglet demonstrated a zone of fibrin and hemorrhage at the apex resection site, shown at 2× magnification in Figure 16A. At the apex resection site, the proliferative mesenchymal cells extend into fibrin (red arrow in Figure 16B at 4× magnification) as well as into a narrow focus of necrotic cardiomyocytes (blue arrow in Figure 16B at 4× magnification). The fibrin and hemorrhage zone was bordered by a dense cellular population of streaming mesenchymal cells that formed a wide margin blending into and insinuating in and amongst existing cardiomyocytes (fibrin zone shown with a red arrow, border zone shown with yellow arrow in Figure 16A). From the 20× magnification images, these cells formed streaming, somewhat fascicular arrays of spindyloid cells with large, plump, variably sized, ovoid to elongated nuclei, and multiple prominent nucleoli (Figure 16C,D). At 40× magnification, mitotic events, where cell division is actively ongoing, were easily identified (green arrows in Figure 16E,F), and coexisting nuclei had fine chromatin and large prominent nucleoli. Some interspersed cells in this proliferative population with similar nuclei had slender strands of cytoplasm with the suggestion of cross striations, resembling immature cardiomyocytes (yellow arrow in Figure 16E).

## 4. Discussion

We chose 0 days and seven days because of the reported mouse apex resection study, which concluded that there was a regenerative response similar to zebrafish in neonatal mice before day 7 after birth [35]. We had to apply partial apex resection instead of the full ventricular apex resection used in the mice study [35,36], which would inevitably result in rapid blood loss from the neonatal pig heart ventricle due to the high ventricular pressure in large mammals. The unfortunate losses of two piglets during our surgical procedure verified that full apex resection was not an option since the small accidental cut into the ventricular chamber led to rapid blood loss. The ventricular pressure and the extent of hemorrhage could not be controlled by hemostatic mechanisms even for a small transmural defect in the piglets, although this was a successful protocol used in the mouse studies and allowed for full apex resection [35,36].

The partial apex resection protocol allowed us to successfully induce surgical injury on 0-day-old and seven-day-old neonatal pig hearts. During surgery, we noted that the 0-day-old heart muscle tissue was more pliable, and the partial apex resection could be more easily performed when compared with the seven-day-old hearts, which had more rigid heart muscle tissue. The underlying reason for the increased heart tissue stiffness and decreased tissue pliability after the piglet’s birth is the rapid deposition and maturation of the collagen network, which binds the heart muscle fibers and forms the cardiomyocyte ECM lacunae [50,51,52]. We also noticed that the apex region had the thinnest wall thickness in the seven-day-old hearts due to anatomical development; therefore, the surgical site had to be moved to the site slightly anterolateral to the apex on the left ventricular free wall. The challenge of operating seven-day-old piglet hearts is somewhat consistent with an observation in mice reported by Porrell et al., who reported that surgical lethality was much higher in P7 pups compared with P1 pups; therefore, a smaller proportion of the apex was resected in P7 pups, with minimal chamber exposition [35].

Table 2 shows that the resected tissue mass was 8.47 ± 11.2 mg for the 0-day-old piglets and 5.62 ± 1.75 mg for the seven-day-old piglets. The larger lesion (8.47 ± 11.2 mg) in 0-day-old piglet hearts, however, still experienced better regeneration when compared to the overall smaller lesion (5.62 ± 1.75 mg) in seven-day-old piglet hearts, which suggests that other factors, such as cells, biofactors, and ECM microenvironment and stiffness, might play more determining roles on the regeneration potential (as well as cardiac function-EF and FS) than the initial wound size. Nevertheless, the piglets responded to the surgery well, recovered rapidly, and lived normally with the mother pig post-surgery (Figure 1C).

The echocardiogram measurements indicated an overall significant decrease (*p* < 0.05) in EF (Figure 2D) and FS (Figure 3D) for the seven-day-old apex resection group over the course of four weeks post-surgery, suggesting that scar formation at the resection site might affect the overall ventricular function. We noticed that, for the 0-day surgery groups, a decreasing trend in EF and FS also occurred at the last two time points (3rd and 4th weeks PO) (Figure 2B and Figure 3B). One possibility might be that while there is cardiomyocyte regeneration, this has not yet led to full functional recovery in these animals compared to the controls. Alternatively, despite evidence of regeneration, differences in the spatial orientation of new myofibrils may result in less efficient apical contractile function.

Our histological study provides a solid observation of the tissue remodeling process after the introduction of the injury. From histological images of the heart explanted at 4W PO, we further noticed that two of the 0-day apex resection group animals (#2034 and #2035), with resected apical mass similar to the average resected mass of the seven-day surgery group (Table 2), had only subtle deviation at the resection area from the surrounding myocardium (Figure 6C,D #2034 and 2035). When compared to the control hearts, these piglets had largely imperceptible changes, including slight disorganization, a slightly higher number of cells, and vascular prominence in the absence of an increased extracellular collagenous matrix. The other piglets in this group, which had a much larger resected apex mass (#2036 and #2037) (Table 2), showed a population of proliferative cells dissecting into adjacent myocardial fibers (Figure 6E,F #2036, #2037). Those cells were reminiscent of cardiomyocytes but were associated with the ECM. However, all piglets in the seven-day-old surgery group, independent of the resected apical mass (Table 2), were found to have moderate to severe fibrosis in the epicardial region as well as the interstitial myocardium (Figure 8C–E). This fibrosis was associated with the excess extracellular collagenous matrix compared to the control piglets in the seven-day group, indicating scar formation. We could hence conclude that the observed differences in regeneration potential were not caused by variations of resected apical mass.

An important finding came from the premature death of one 0-day apex resection piglet (briefly lain on and accidentally killed by the mother sow three days PO; necropsied in fresh condition). At the lesion site, this piglet had a fibrin and hemorrhage zone (Figure 16A, red arrow), bordered by a wide margin of dense, streaming mesenchymal cells (Figure 16A, yellow arrow), which then blended into existing cardiomyocytes. The active cardiac regeneration process was evidenced clearly by the following histological observation: (i) the streaming mesenchymal cells had fascicular arrays of spindyloid cells with large, plump, variably sized nuclei and multiple prominent nucleoli (Figure 16C–F); (ii) active mitotic events were observed (Figure 16E,F, green arrow); (iii) the proliferative mesenchymal cells extended into fibrin (Figure 16A,B, red arrow) and the local focus of necrotic cardiomyocytes (Figure 16B, blue arrow); some interspersed cells in the proliferative population had similar nuclei and slender strands of cytoplasm with the suggestion of cross striations, reminiscent of immature cardiomyocytes (Figure 16E,F, yellow arrow). In addition, the pattern of proliferative cells lacked the histologic features of fibrovascular/granulation tissue as might precede maturation to scar tissue (Figure 13A,B).

A unique contribution of our study is the assessment of the regenerative potential in neonatal pig hearts using an apex resection model, which can be directly compared with the zebrafish apex resection and the neonatal mice apex resection studies [33,34,35,36,37]. Our study provides morphologically convincing pathological observations on the neonatal hearts post-injury via a traditional diagnostic approach. Three major observations are made in our research: (1) The piglets lose the full regenerative potential by seven days after birth but preserve the regenerative potential of resected apex tissue within one day of birth; (2) in the 0-day surgery group, the cardiac regeneration is associated with proliferative mesenchymal cells bordering the fibrin and hemorrhage zone which differentiate into immature cardiomyocytes; (3) in the seven-day surgery group, scar formation predominates at the resection site, exhibiting epicardial fibrosis along with moderate to severe dissecting interstitial fibrosis.

The transient regeneration potential we observed is consistent with a recent publication by Zhu et al., which reported that the neonatal pig heart was capable of regeneration following acute myocardial infarction (created by permanent ligation of the left descending coronary artery) during the first days of life [42]. Zhu et al. also pointed out that the regeneration observed in their acute myocardial infarction model was associated with the induction of cardiomyocyte proliferation and was lost when cardiomyocytes exit the cell cycle shortly after birth [42]. A similar conclusion was also reached in a publication by Ye et al. [43], which again created an acute myocardial infarction by permanent ligation of the left descending coronary artery. Ye et al. reported that two-day-old neonatal pigs can regenerate functional cardiac muscle by repopulating the infarct with newly derived cardiomyocytes in the absence of fibrosis, and this capacity is lost thereafter [43].

Our morphologically convincing observations showed that, in the 0-day apex resection heart, there were dense, proliferative mesenchymal cells in the bordering regions of the fibrin and hemorrhage zone, and those cells exhibited differentiation characteristics of immature cardiomyocytes. Morphologically, mitoses were visible in cells with overt features of cardiomyocytes including apparent cross striations and cytoplasm similar to sarcoplasm. However, the origin of those proliferative mesenchymal cells is not clear yet in the current study. The mouse apex resection model by Porrello et al. suggested that the regenerative response post-partum was mediated via proliferation of the remaining uninjured cardiomyocytes, which lost this regenerative potential when exiting the cell cycle by postnatal day 7 [35]. Both porcine acute MI models via permanent ligation of coronary artery by Zhu et al. [42] and Ye et al. [43] also indicated that cardiac regeneration observed in day 1 and day 2 animals might be largely attributable to the proliferation of preexisting cardiomyocytes; however, due to the inability to track cell lineages in their porcine MI models, both studies did not rule out possible contribution from stem cell-mediated myogenesis or other potential mechanisms [42,43].

The importance of this topic keeps attracting research efforts across the world. A recent conference proceeding by Malliaras et al. reported that they did not observe a robust cardiac regenerative response in neonatal porcine hearts post-MI [44]. Using a similar protocol (permanent ligation of the left descending coronary artery), Malliaras noted that hearts of both one-day-old and three-day-old neonatal pigs exhibited substantial scarring and significant hypokinesia of the infarcted myocardium post-MI [44]. Validating investigative findings is an important task for researchers. We believe that, with more experiments and mechanistic investigations on the way, the understanding of the transient regenerative potential of cardiac muscle at the neonatal stage will undoubtedly be more thorough and complete.

## 5. Conclusions

In this study, we successfully performed a partial apex resection surgery on 0-day-old and seven-day-old neonatal piglets and used this model to assess the regenerative potential of the apex resection site, which allows a direct comparison with the zebrafish apex resection and the neonatal mice apex resection studies [33,34,35,36,37]. Our qualitative findings suggest that the piglets lose the full myocardial regenerative potential by seven days after birth but greatly preserve the regenerative potential within one day post-partum. Upon gross necropsy, for piglets euthanized four weeks post-surgery, all 0-day-old hearts showed no signs of scarring or any indication of the induced injury. Compared with the 0-day surgery group, the seven-day surgery group experienced a decrease in ejection fraction and fractional shortening. Our histological analysis of the explants 4W PO revealed that the piglet hearts from the 0-day surgery group exhibited various degrees of regeneration, with half of the hearts demonstrating full regeneration and the other half demonstrating partial regeneration. In contrast, each piglet heart from the seven-day surgery group showed epicardial fibrosis along with moderate to severe dissecting interstitial fibrosis, revealing scar formation in the resection site and a loss of full regenerative potential.

When assessing the progression of repair, we found that, for the 0-day surgery piglets euthanized at 5D PO and 1W PO, half had signs of proliferating mesenchymal cells that demonstrated active regeneration, while the other half had increased extracellular collagen. The seven-day surgery piglets euthanized at 5D PO, and 1W PO showed evidence of greatly increased extracellular collagen, while some piglets showed proliferating mesenchymal cells, implicating a regenerative effort is ongoing while scar formation predominates. We further observed that in one 0-day apex resection heart obtained three days post-surgery, there were dense, proliferative mesenchymal cells in the bordering regions of the fibrin and hemorrhage zone, and those cells exhibited differentiation characteristics of immature cardiomyocytes.

While our histological assessment did provide evidence of cell proliferation by morphology-based features such as active cell mitosis, cell nuclei and nucleoli, and cell density and alignment in the repairing region of the 0-day partial apex resection surgery group (Figure 12 and Figure 16), a major limitation is that we did not specifically assess cell proliferation and phenotype in this study. These assessments would indeed be critical for our next steps in order to provide irrefutable evidence of cell phenotypes and their proliferative responses. Further investigations are hence warranted to determine the underlying mechanisms of heart regeneration during the neonatal stage, which will focus on biochemical and biological factors, cell phenotypes, cellular behavior, ECM remodeling, and mechanical and structural microenvironments. With the obtained knowledge, researchers can harness the biological and biomechanical cues that trigger full heart muscle regeneration and help design novel therapies to assist myocardial regeneration in MI patients.

Lastly, for the seven-day surgery group in our study, there is clear evidence of scar formation rather than cardiac regeneration, i.e., scar formation predominates, and the resection site exhibits epicardial fibrosis along with moderate to severe dissecting interstitial fibrosis. Given that our findings indirectly indicate that scar formation predominates and surpasses the activities of cardiac regeneration even at the early developmental stage (seven days old), a major goal for improving future cardiac repair outcomes should, therefore, emphasize the minimization of scar tissue formation, in addition to efforts to promote cardiomyocyte regeneration. In other words, an important focus of future investigations should be the reduction of scar tissue formation, which remains a significant challenge in cardiac repair.

## Figures and Tables

**Table 1:** Experimental layout for assessing the regenerative potential of neonatal pig hearts.

Animal Numbers and Time Points for Heart Harvest			
Total N = 36	Heart Harvest 5 Day Post-Surgery	Heart Harvest 1 Week Post-Surgery	Heart Harvest 4 Weeks Post-Surgery
Apex Removal Surgery on 0-day-old Piglets	N = 4	N = 4	N = 5
Apex Removal Surgery on 7-day-old Piglets	N = 5	N = 5	N = 5
Sham Surgery on 0-day-old Piglets	NA	N = 2	N = 2
Sham Surgery on 7-day-old Piglets	NA	N = 2	N = 2

**Table 2:** Surgery information for each piglet. The table summarizes sex, body weight, resected tissue mass, and age of the piglets.

**0 Day Control and 0 Day Surgery**				
**Piglet ID**	**Sex of Piglet**	**Body Weight at Surgery (lbs)**	**Tissue Mass Removed from Apical Region (mg)**	**Age at Time of Apical Resection**
2033	M	3.31	0 day control	NA
2038	F	2.81	0 day control	NA
005	F	3.53	0 day control	NA
017	M	2.20	0 day control	NA
2034	M	4.31	*, sample lost in pig (cut similar to 2035)	0 days
2035	F	3.31	5.1	0 days
2036	M	3.69	22.6	0 days
2039	M	3.79	34.5 (accidently killed by the mother sow 3 days PO)	0 days
2037	F	3.56	*, sample lost in pig (cut similar to 2039)	0 days
004	M	3.09	2.3	0 days
013	F	3.00	2.2	0 days
014	M	4.37	16.8	0 days
015	F	4.10	3.1	0 days
001	F	3.60	2.9	0 days
002	F	3.75	2.6	0 days
018	F	3.53	0.8	0 days
024	M	3.09	0.3	0 days
**AVG ± STD**		**3.64 ± 0.48**	**8.47 ± 11.20**	
**7 Day Control and 7 Day Surgery**				
**Piglet ID**	**Sex of Piglet**	**Body Weight at Surgery (lbs)**	**Tissue Mass Removed from Apical Region (mg)**	**Age at Time of Apical Resection**
2071	F	6.31	7 day control	NA
2077	M	3.42	7 day control	NA
012	F	5.51	7 day control	NA
027	M	6.59	7 day control	NA
2073	M	3.09	13.0	7 days
2075	M	4.41	6.0	7 days
2076	M	7.48	8.4	7 days
2078 *	M	4.86	*, heart tore during surgery; euthanized-massive blood loss	7 days
2079 *	F	5.29	*, resected into ventricle; euthanized-massive blood loss	7 days
007	F	5.07	6.7	7 days
008	M	5.07	5.2	7 days
029	F	7.74	12.2	7 days
031	M	7.61	2.3	7 days
009	M	5.07	9.9	7 days
010	F	6.17	7.3	7 days
011	M	5.07	2.4	7 days
016	F	6.72	11.5	7 days
026	M	7.34	6.5	7 days
028	M	8.07	9.0	7 days
**AVG ± STD**		**6.39 ± 1.19**	**5.62 ± 1.75**	

Note: * represents data not available.

**Table 3:** Body weight, weight of the heart, and age of piglets at euthanasia. * denotes the loss of animal during the surgery or experiment.

Piglet ID and Group	Body Weight at Euthanasia (lbs)	Heart Mass at Euthanasia (g)	Age at Euthanasia
2033 (0 day control)	13.28	35.73	4 weeks
2038 (0 day control)	13.88	56.70	4 weeks
**AVG ± STD**	**13.58 ± 0.30**	**46.22 ± 10.49**	**----------**
2034 (0 day surgery)	14.52	42.52	4 weeks
2035 (0 day surgery)	15.40	39.69	4 weeks
2036 (0 day surgery)	15.66	42.52	4 weeks
2037 (0 day surgery)	16.24	48.19	4 weeks
**AVG ± STD**	**15.46 ± 0.62**	**43.23 ± 3.09**	**----------**
2039 * (0 day surgery)	-----------------------	------------------	Lost at 3 days
2071 (7 day control)	16.78	51.03	5 weeks
2077 (7 day control)	16.82	48.19	5 weeks
**AVG ± STD**	**16.80 ± 0.02**	**49.61 ± 1.42**	**----------**
2073 (7 day surgery)	9.48	25.51	5 weeks
2075 (7 day surgery)	11.16	31.18	5 weeks
2076 (7 day surgery)	21.52	56.70	5 weeks
**AVG ± STD**	**14.05 ± 5.32**	**37.80 ± 13.57**	**----------**
2078 * (7 day surgery)	-----------------------	------------------	Lost during surgery
2079 * (7 day surgery)	-----------------------	------------------	Lost during surgery
005 (0 day control)	5.82	26.48	1 week
017 (0 day control)	4.08	18.92	1 week
**AVG ± STD**	**4.95 ± 0.87**	**22.70 ± 3.78**	**----------**
004 (0 day surgery)	4.02	14.83	5 days
013 (0 day surgery)	4.23	17.67	5 days
014 (0 day surgery)	6.48	26.73	5 days
015 (0 day surgery)	5.86	28.00	5 days
**AVG ± STD**	**5.15 ± 1.05**	**21.81 ± 5.67**	**----------**
001 (0 day surgery)	5.46	20.01	1 week
002 (0 day surgery)	5.30	22.82	1 week
018 (0 day surgery)	5.86	34.91	1 week
024 (0 day surgery)	5.20	28.57	1 week
**AVG ± STD**	**5.46 ± 0.25**	**26.57 ± 5.72**	**----------**
012 (7 day control)	10.10	31.16	2 week
027 (7 day control)	9.88	31.52	2 week
**AVG ± STD**	**9.99 ± 0.11**	**31.34 ± 0.18**	**----------**
007 (7 day surgery)	7.64	24.71	12 days
008 (7 day surgery)	6.92	22.22	12 days
011 (7 day surgery)	9.06	39.72	12 days
029 (7 day surgery)	7.74	34.27	12 days
031 (7 day surgery)	7.61	34.14	12 days
**AVG ± STD**	**7.79 ± 0.70**	**31.01 ± 6.53**	**----------**
009 (7 day surgery)	8.30	34.54	2 week
010 (7 day surgery)	10.34	34.36	2 week
016 (7 day surgery)	6.72	37.80	2 week
026 (7 day surgery)	7.34	42.16	2 week
028 (7 day surgery)	8.07	44.11	2 week
**AVG ± STD**	**8.15 ± 1.23**	**38.59 ± 3.95**	**----------**

## Supplementary Materials

The following supporting information can be downloaded at: https://www.mdpi.com/article/10.3390/bioengineering9080401/s1, Table S1: Experimental layout for assessing the regenerative potential of neonatal pig hearts; Table S2: Surgery information for each piglet. Table summarizes sex, body weight, resected tissue mass, and age of the piglets; Table S3: Body weight, weight of the heart, and age of piglets at euthanasia.
