# Supplementary material for: Investigating the Transient Regenerative Potential of Cardiac Muscle Using a Neonatal Pig Partial Apical Resection Model
Source: Bioengineering (Basel). 2022 Aug 18;9(8):401. doi: 10.3390/bioengineering9080401 (PMC9404987; doi:10.3390/bioengineering9080401)
Supplement: Supplementary file 1 [file bioengineering-09-00401-s001.zip › bioengineering-1689465-supplementary.pdf]

Supplemental Tables

Supplemental Table S1. Experimental layout for assessing the regenerative potential of neonatal pig hearts.

Total N=36		
Age Group	0 Day Old Piglet	7 Day Old Piglet
Apex Removal Surgery	N=13	N=15
Sham Surgery (Control)	N=4	N=4
Heart Harvest for Assessment		
Apex Removal Surgery	N=4 (5 Days PO)	N=5 (5 Days PO)
	N=4 (1 Week PO)	N=5 (1 Week PO)
	N=5 (4 Weeks PO)	N=5 (4 Weeks PO)
Sham Surgery	N=2 (1 Week PO)	N=2 (1 Week PO)
	N=2 (4 Weeks PO)	N=2 (4 Weeks PO)

Supplemental Table S2: Surgery information for each piglet. Table summarizes sex, body weight, resected tissue mass, and age of the piglets.

Piglet ID	Sex of Piglet	Body Weight at Surgery (lbs)	Tissue Mass Removed from Apical Region (mg)	Age at Time of Apical Resection
2033	M	3.31	0 day control	NA
2038	F	2.81	0 day control	NA
005	F	3.53	0 day control	NA
017	M	2.20	0 day control	NA
2034	M	4.31	*, sample lost in pig (cut similar to 2035)	0 days
2035	F	3.31	5.1	0 days
2036	M	3.69	22.6	0 days
2039	M	3.79	34.5 (accidently killed by the mother sow 3 days PO)	0 days
2037	F	3.56	*, sample lost in pig (cut similar to 2039)	0 days
004	M	3.09	2.3	0 days
013	F	3.00	2.2	0 days
014	M	4.37	16.8	0 days
015	F	4.10	3.1	0 days
001	F	3.60	2.9	0 days
002	F	3.75	2.6	0 days
018	F	3.53	0.8	0 days
024	M	3.09	0.3	0 days
**AVG ± STD**		**3.64 ± 0.48**	**8.47 ± 11.20**	
2071	F	6.31	7 day control	NA
2077	M	3.42	7 day control	NA
012	F	5.51	7 day control	NA
027	M	6.59	7 day control	NA
2073	M	3.09	13.0	7 days
2075	M	4.41	6.0	7 days
2076	M	7.48	8.4	7 days
2078*	M	4.86	*, heart tore during surgery; euthanized-massive blood loss	7 days
2079*	F	5.29	*, resected into ventricle; euthanized-massive blood loss	7 days
007	F	5.07	6.7	7 days
008	M	5.07	5.2	7 days
029	F	7.74	12.2	7 days
031	M	7.61	2.3	7 days
009	M	5.07	9.9	7 days
010	F	6.17	7.3	7 days
011	M	5.07	2.4	7 days
016	F	6.72	11.5	7 days
026	M	7.34	6.5	7 days
028	M	8.07	9.0	7 days
**AVG ± STD**		**6.39 ± 1.19**	**5.62 ± 1.75**	

Supplemental Table S3: Body weight, weight of the heart, and age of piglets at euthanasia.

Piglet ID and Group	Body Weight at Euthanasia (lbs)	Heart Mass at Euthanasia (g)	Age at Euthanasia
2033 (0 day control)	13.28	35.73	4 weeks
2038 (0 day control)	13.88	56.70	4 weeks
**AVG ± STD**	**13.58 ± 0.30**	**46.22 ± 10.49**	----------
2034 (0 day surgery)	14.52	42.52	4 weeks
2035 (0 day surgery)	15.40	39.69	4 weeks
2036 (0 day surgery)	15.66	42.52	4 weeks
2037 (0 day surgery)	16.24	48.19	4 weeks
**AVG ± STD**	**15.46 ± 0.62**	**43.23 ± 3.09**	----------
2039* (0 day surgery)	----------------------	------------------	Lost at 3 days
2071 (7 day control)	16.78	51.03	5 weeks
2077 (7 day control)	16.82	48.19	5 weeks
**AVG ± STD**	**16.80 ± 0.02**	**49.61 ± 1.42**	----------
2073 (7 day surgery)	9.48	25.51	5 weeks
2075 (7 day surgery)	11.16	31.18	5 weeks
2076 (7 day surgery)	21.52	56.70	5 weeks
**AVG ± STD**	**14.05 ± 5.32**	**37.80 ± 13.57**	----------
2078* (7 day surgery)	----------------------	------------------	Lost during surgery
2079* (7 day surgery)	----------------------	------------------	Lost during surgery
005 (0 day control)	5.82	26.48	1 week
017 (0 day control)	4.08	18.92	1 week
**AVG ± STD**	**4.95 ± 0.87**	**22.70 ± 3.78**	----------
004 (0 day surgery)	4.02	14.83	5 days
013 (0 day surgery)	4.23	17.67	5 days
014 (0 day surgery)	6.48	26.73	5 days
015 (0 day surgery)	5.86	28.00	5 days
**AVG ± STD**	**5.15 ± 1.05**	**21.81 ± 5.67**	----------
001 (0 day surgery)	5.46	20.01	1 week
002 (0 day surgery)	5.30	22.82	1 week
018 (0 day surgery)	5.86	34.91	1 week
024 (0 day surgery)	5.20	28.57	1 week
**AVG ± STD**	**5.46 ± 0.25**	**26.57 ± 5.72**	----------
012 (7 day control)	10.10	31.16	1 week
027 (7 day control)	9.88	31.52	1 week
**AVG ± STD**	**9.99 ± 0.11**	**31.34 ± 0.18**	----------
007 (7 day surgery)	7.64	24.71	5 days
008 (7 day surgery)	6.92	22.22	5 days
011 (7 day surgery)	9.06	39.72	5 days
029 (7 day surgery)	7.74	34.27	5 days
031 (7 day surgery)	7.61	34.14	5 days
**AVG ± STD**	**7.79 ± 0.70**	**31.01 ± 6.53**	----------
009 (7 day surgery)	8.30	34.54	1 week
010 (7 day surgery)	10.34	34.36	1 week
016 (7 day surgery)	6.72	37.80	1 week
026 (7 day surgery)	7.34	42.16	1 week
028 (7 day surgery)	8.07	44.11	1 week
**AVG ± STD**	**8.15 ± 1.23**	**38.59 ± 3.95**	----------
